# The Biomechanics of Fibrillin Microfibrils: Lessons from the Ciliary Zonule

**DOI:** 10.3390/cells13242097

**Published:** 2024-12-18

**Authors:** Pooja Rathaur, Juan Rodriguez, John Kuchtey, Samuel Insignares, Wendell B. Jones, Rachel W. Kuchtey, Steven Bassnett

**Affiliations:** 1 Department of Ophthalmology & Visual Sciences, Washington University School of Medicine, St. Louis, MO 63110, USA; poojar@wustl.edu (P.R.);; 2 Department of Basic Sciences, University of Health Sciences and Pharmacy in St. Louis, St. Louis, MO 63110, USA; juan.rodriguez@uhsp.edu; 3 Vanderbilt Eye Institute, Department of Ophthalmology & Visual Sciences, Vanderbilt University Medical Center, Nashville, TN 37232, USA; john.kuchtey@vumc.org (J.K.); samuel.insignares@vumc.org (S.I.); rachel.w.kuchtey@vumc.org (R.W.K.); 4 Department of Molecular Physiology and Biophysics, Vanderbilt University Medical Center, Nashville, TN 37232, USA

**Keywords:** zonule, microfibril, Marfan syndrome, fibrillin

## Abstract

Marfan syndrome is an inherited connective tissue disorder that affects the cardiovascular, musculoskeletal, and ocular systems. It is caused by pathogenic variants in the fibrillin-1 gene (*FBN1*). Fibrillin is a primary component of microfibrils, which are found throughout the extracellular matrix (ECM) and provide elasticity and resilience to connective tissue. Microfibrils also play a role in signaling by sequestering growth factors and interacting with cell surface receptors. In many tissues, microfibrils are interwoven with elastin, collagens, and other elements of the ECM. However, uniquely in the ciliary zonule of the eye, microfibrils exist in cell-free bundles largely devoid of other components. This structure offers a rare opportunity to study a pure population of fibrillin microfibrils in a relatively native state. Here, we briefly review the organization of the zonule and describe recent experiments in which we measure zonular biomechanics, providing insights into microfibril dynamics that would be challenging to obtain in other contexts.

## 1. Introduction

Marfan syndrome (MFS; MIM #154700) is a connective tissue disorder caused by pathogenic variants in the *FBN1* gene [1], which encodes fibrillin-1, a key component of the extracellular matrix (ECM). MFS affects approximately 1:5000 individuals [2] and is characterized by pleiotropic impacts on the cardiovascular, musculoskeletal, and ocular systems [2,3].

Fibrillin-1 is a large (≈350 kDa) cysteine-rich glycoprotein with the modular domain structure characteristic of the fibrillin family [4]. Most of the protein comprises tandemly arranged epidermal growth factor-like (EGF) domains, 43 of which contain calcium-binding sequences (cbEGF). The cbEGF domains are interspersed by seven transforming growth factor β (TGFβ)-binding protein-like domains (also known as eight-cysteine domains) and two hybrid domains that combine elements of both the cbEGF and TGFβ domains.

Fibrillin assembles into long polymers that constitute the main structural element of microfibrils [5], which are essential components of the ECM, lending both resiliency and elasticity to connective tissues. Electron microscopy reveals that microfibrils are 10–12 nm wide and have a distinctive beaded appearance with a periodicity of approximately 56 nm. Each repetitive unit consists of a bead, an arm region, an inter-bead region, and a shoulder region [6].

Structural studies indicate that the periodic structure likely comprises eight (pseudo)symmetrically arranged fibrillin-1 monomers [7]. When linearized, fibrillin has a length of about 150 nm. Understanding how a molecule of this size might be accommodated in a periodic 56 nm structure has been challenging. Both staggered and unstaggered models have been proposed (reviewed in [8]). Still, recent Cryo-EM studies support a linear head-to-tail model, where the N- and C-termini of fibrillin monomers interact within the crowded interior of the bead region [6].

The elastic properties of individual microfibrils have been measured using molecular combing [9], providing an estimate for the elastic modulus of about 100 MPa. Interestingly, this is considerably higher than the values determined for bundled microfibrils [10,11,12]. X-ray diffraction studies on microfibrils under tension indicate surprisingly little change in bead periodicity with strain. At 50% strain, for example, the bead-to-bead distance increases by only 3% [13,14]. Therefore, the detailed relationship between the molecular structure of fibrillin microfibrils and their elastic properties remains poorly understood.

Microfibrils play critical mechanical roles, but they also bind and localize bioactive proteins. Notably, they are a rich source of latent TGFβ ligands, sequestered via interactions with latent TGFβ-binding proteins (LTBPs) [15]. Additionally, bone morphogenetic proteins (BMPs), which are also members of the TGFβ superfamily, associate directly with microfibrils [16]. Hence, microfibrils serve both structural and signaling functions.

In many tissues, microfibrils contribute to composite structures known as elastic fibers, which are prominent components of the ECM. Elastic fibers typically consist of an amorphous elastin core surrounded by a loose cladding of microfibrils. Elastic fibers allow tissues to stretch and recoil, which are essential properties in settings such as the walls of large arteries, the lungs, ligaments, and skin.

Less commonly, fibrillin microfibrils exist independently of elastin, with the ciliary zonule being a well-known example. This suspensory ligament connects the ocular lens to the ciliary body, located on the eye’s inner wall. It is composed entirely of fibrillin microfibrils, bundled into fibers 1–50 μm in diameter. Because the zonule is an accessible, macroscopic, cell-free structure, it serves as an excellent model for studying the composition, structure, and biomechanical properties of microfibrils. Several comprehensive reviews have discussed the organization and function of the ciliary zonule [17,18,19].

The revised Ghent nosology outlines the diagnostic criteria for MFS [20]. Among these criteria, ectopia lentis (a displaced lens due to compromised zonular fibers) and aortic root aneurysm are the two cardinal features. Ocular complications are common in MFS patients [21] and contribute significantly to the treatment burden [22]. In addition to ectopia lentis (which affects approximately half of all MFS patients [23,24]), individuals with MFS may experience early-onset cataracts, a flattened cornea [25], increased axial length, and refractive errors (usually myopia, but occasionally hyperopia) [26]. Furthermore, MFS patients face a significantly higher risk of blindness due to conditions such as rhegmatogenous retinal detachment [27] and glaucoma [28].

In this article, we briefly review the structure and composition of the ciliary zonule before delving into the findings of recent biomechanical studies we have conducted on genetically modified mice. These studies offer insights into the relationship between the molecular composition of microfibrils and their mechanical properties and are pertinent to the MFS phenotype.

## 2. Anatomical Organization of the Ciliary Zonule

**Figure 1:**
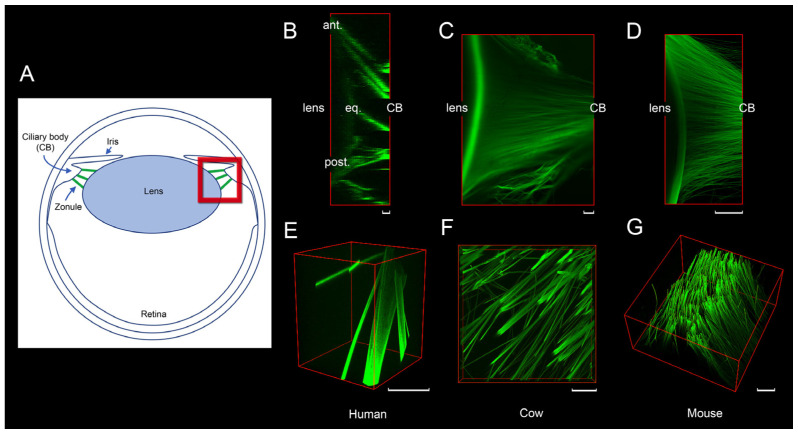
Organization of the ciliary zonule in human, cow, and mouse eyes. A schematic diagram (**A**) illustrates the internal structure of the eye in the sagittal plane. Using previously published methods [32], zonular fibers (green) were visualized extending from the ciliary body to the lens equator. The boxed region (red) indicates the approximate area from which the images in panels (**B**–**G**) were captured. Confocal image stacks were collected from samples stained with a fluorescent lectin (*Lens culinaris* agglutinin) and reconstructed using volume visualization software (Huygens, 24.10; SVI) to generate sagittal (**B**–**D**) or oblique (**E**,**F**) views of the zonule. In humans (**B**,**E**), the zonule consists of thick but relatively sparse fibers organized into distinct anterior (ant.), equatorial (eq.), and posterior (post.) groupings. In contrast, the cow (**C**,**F**) and mouse (**D**,**G**) zonules consist of a uniform meshwork of fine fibers. Scale bars = 50 μm.

The vertebrate camera eye contains a centrally positioned lens suspended from the inner eyewall by a ligamentous structure. In mammals, this structure consists of radially oriented fibers known collectively as the ciliary zonule, which center and stabilize the lens (Figure 1). A similar structure is present in the avian eye [29], while in lampreys and teleosts, a transparent membrane or fibrillar bundles, respectively, serve this purpose [30,31].

In humans and other primates, the focal length of the youthful lens can be adjusted to focus on near objects, providing an accommodative range of 10 or more diopters [33], although this facility wanes with age. To change the shape of the lens the zonular fibers transmit forces generated by the ciliary muscle. Thus, in addition to centering the lens, the fibers of the human zonule are an essential component of the dynamic focusing mechanism.

Perhaps reflecting this more active role, the zonular fibers of the human eye (Figure 1B,E) are considerably thicker than those in species with limited or no accommodation, such as cows (Figure 1C,F) and mice (Figure 1D,G).

In mammals, the zonule extends from the surface of the non-pigmented ciliary epithelium (NPCE) and terminates at anchorage points that span the lens equator. At their proximal ends, quick-freeze deep-etch images reveal that zonular fibers merge seamlessly with elements of the inner limiting membrane, the basement membrane of the NPCE [34]. The zonular fibers are thickest near their origin and usually branch, often multiple times, as they approach the lens capsule [35], attaching to a layer of superficial microfibrils known as the zonula lamella. Microfibrils of the zonular lamella are arranged meridionally, forming a girdle-like band that encircles the lens equator.

The fibers arise from a relatively narrow region of the ciliary epithelium and terminate on the much broader zonula lamella. As a result, in all species, fibers diverge as they approach the lens (see Figure 1).

**Figure 2:**
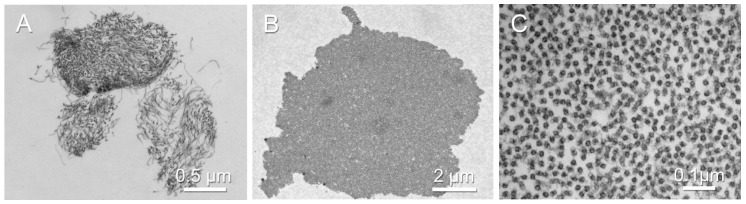
Transmission electron micrographs of cross-sectioned zonular fibers prepared and imaged as described [32] showing the constituent microfibrils. (**A**) Transverse view of three zonular fibers in the mouse eye. (**B**) A single cross-sectioned human zonular fiber. (**C**) Individual cross-sectioned microfibrils in the interior of a human zonular fiber.

The number of zonular fibers that suspend the lens is not directly correlated with eye size. For instance, zonular fibers are numerous in the large eyes of cows (Figure 1C) and the much smaller eyes of mice (Figure 1D). Detailed analysis in mice suggests that as many as 30,000 fibers may be present [32]. In contrast, despite its larger diameter (9 mm vs. 2.5 mm), the adult human lens is suspended by only a few hundred fibers [36], each of which is more than tenfold thicker than fibers in the mouse zonule (see Figure 1 and Figure 2).

In all species, the thickness of adjacent zonular fiber varies considerably. In humans, for example, fiber diameters range from a few micrometers to over 20 μm (Figure 1E). In contrast, mouse zonular fibers rarely exceed 1.5 μm in thickness, with a reported modal value of 0.5–0.6 μm [32].

Transverse sections of mouse or human zonular fibers, examined under an electron microscope, reveal thousands of cross-sectioned microfibrils (Figure 2A,B). Individual microfibrils have circular cross-sections, approximately 12 nm in diameter, with electron-dense rims and electron-lucent cores (Figure 2C).

In cross-sectioned human zonular fibers, the microfibrils are present at densities of around 1200 microfibrils per μm^2^. A single 5 μm-wide fiber would contain about 24,000 microfibrils at this density. If 12 nm-wide microfibrils were arranged in a close-packed hexagonal arrangement (the most efficient pattern, filling about 91% of the available area), 158,000 microfibrils could fit in a 5 μm-wide zonular fiber. Thus, the observed packing density is only about 15% of what is geometrically possible.

It is highly likely that the packing density of microfibrils in the interior of a zonular fiber changes when the fiber is placed under tension. However, Poisson’s ratio (the ratio between transverse and axial strain) has not yet been determined for a zonular fiber or its constituent microfibrils. Thus, predicting the degree to which microfibril density will increase during fiber elongation remains difficult.

The composition of the material that fills the spaces between neighboring microfibrils is uncertain. In situ, the fibers are surrounded and likely permeated by aqueous humor, an ultrafiltrate of blood plasma with a relatively low protein content. It is also possible that polysaccharides coat or pervade the fibers. Although standard histochemical preparation techniques do not well preserve sugar structures, evidence suggests the presence of chondroitin sulfate proteoglycans and heparan sulfate proteoglycans in the zonule [37], potentially complexed with hyaluronic acid [38]. The fibers are also intensely labeled by lectins (see Figure 1), which are molecules that bind carbohydrates with high affinity. The properties of the interstitial material are important because the movement of fluid in or out of zonular fibers under tension could significantly influence their viscoelastic properties (see Section 5).

## 3. Composition of the Zonule

The zonule is a cell-free structure, and with careful dissection, zonular fibers can be directly extracted from the eye, providing a relatively pure sample of microfibrils. Because the zonule is located adjacent to the anterior face of the vitreous (also known as the anterior hyaloid), contamination of the sample with vitreous proteins is difficult to avoid. Additionally, since the globe must be opened to dissect the zonular fibers, there is a risk of trace contamination from the blood. Despite these challenges, several studies have successfully harvested the fibers and used mass spectroscopic methods to define the composition of the zonule, leading to the emergence of a tentative proteome [39,40,41].

The main protein components of the human and bovine zonule are fibrillin-1 (FBN1), latent TGFβ-binding protein-2 (LTBP2), and microfibril-associated protein-2 (MFAP2, sometimes called MAGP1), with fibrillin-1 alone accounting for approximately 70% of the zonular mass. Fibrillin-2, the dominant fibrillin isoform during early development [42,43], remains a significant component of the zonule into adulthood. Given their abundance, these proteins likely serve as essential structural elements and appear to be synthesized primarily by NPCE cells [44]. In addition, several dozen other proteins, each generally comprising 1% or less of the total, contribute to the zonular proteome. Interestingly, despite it being nominally elastin-free, the zonule contains significant levels of lysyl oxidase-like 1 (LOXL1) [40,45], a cross-linking enzyme associated with elastin deposition [46].

Although zonular fibers have variable thickness (Figure 1E), they have an otherwise uniform appearance. Their composition, however, appears to vary depending on their location within the zonule and the age of the organism. For example, MFAP2 is more abundant in the anterior and posterior fibers than in equatorial fibers in mice [32]. Similarly, LTBP2 synthesis does not begin until a week or more after birth in mice, implying that zonular fibers produced before that time may lack LTBP2 [32].

As noted earlier, like the rest of the body, the eye undergoes a developmental switch from *FBN2* to *FBN1* expression. Thus, fibers synthesized early in life are likely enriched in fibrillin-2 [43,44]. In adult zonular fibers, fibrillin-2-rich microfibrils are concentrated in the interior of the fibers, while fibrillin-1-rich microfibrils are more abundant in the outer layers [32]. This observation supports an accretive growth pattern, wherein newly synthesized fibrillin-1-rich microfibrils are added at the fiber surface.

## 4. Synthesis and Turnover

The first signs of the zonule appear during embryonic development while the lens equator is still in direct contact with the inner wall of the optic cup. At this point, *FBN2* and *FBN1* mRNA are detectable in cells near the anterior lip of the optic cup, a region that will eventually differentiate into the ciliary epithelium [43]. By birth, fibrillin-1 and MFAP2 proteins are present in the virtual space between the two tissues. In mice, the lens and optic vesicle begin to separate toward the end of the first postnatal week. At this point, zonular fibers are already visible, spanning the newly formed circumlental space.

Fibrillin-1, the primary component of the zonule, is considered a highly stable protein, with a half-life measured in decades, comparable to that of elastin [47]. This raises the question of whether fibrillin-1 and other zonular proteins undergo significant turnover after their initial synthesis. In situ hybridization studies suggest that NPCE cells continue to transcribe *FBN1* until at least one year of age in mice [44] and up to 35 months in guinea pigs [48]. Additionally, single-cell RNA sequencing of human ocular samples shows strong and sustained expression of *FBN1*, *LTBP2*, and *MFAP2* by NPCE cells in adult eyes [49]. Collectively, these findings suggest continuous microfibril production. Supporting this idea, biomechanical measurements in mice (see Section 5) show a gradual increase in zonular tensile strength with age, implying the slow, ongoing incorporation of new microfibrils into the adult zonule. Direct testing via pulse-chase experiments would be needed to confirm this hypothesis.

Recently, Adamts10 (ADAM Metallopeptidase with Thrombospondin Type 1 Motif 10), a zinc-dependent metalloproteinase, was implicated in the turnover of ocular microfibrils. Mice homozygous for either the *S236X* [50] or *G661R* [51] mutations in *ADAMTS10* develop phenotypes resembling those of human patients with Weill–Marchesani Syndrome 1 (WMS1, an autosomal dominant condition caused by mutations in *ADAMTS10*). Like human patients, these animals exhibit hypermuscularity, thick skin, short stature, and ocular defects. Regarding the ciliary zonule, the zonular fibers in mutant mice are thicker than usual and stain more intensely for fibrillin-2. The accumulation of fibrillin-2-rich microfibrils suggests that the presence of mutant Adamts10 either promotes the production of fibrillin-2 microfibrils, inhibits their degradation, or possibly both [50]. Subsequent studies utilizing *ADAMTS10*-null mice have confirmed fibrillin-2 as a bona fide substrate for Adamts10 [52].

Activation of Adamts10 protease requires the removal of the propeptide by furin convertase [53], a relatively inefficient process. Consequently, only a small fraction of the available Adamts10 enzyme is active. Uncleaved Adamts10 promotes the formation of fibrillin-1 microfibrils, at least in vitro [53]. Thus, Adamts10 appears to play a crucial role in regulating the stoichiometry of fibrillin isoforms, promoting the synthesis of fibrillin-1 in the zonule while concurrently degrading fibrillin-2.

## 5. Biomechanical Properties

The strength and resiliency of zonular fibers are compromised in several human diseases. In these cases, the structural failure of the zonule is readily apparent when examining the eye. When the lens is physically unstable, it indicates that some or all of the zonular fibers have ruptured. Clinically, this manifests as frank lens subluxation or as tremulousness of the lens, referred to as phacodonesis. Since the iris is supported by the anterior lens surface, it too can become destabilized when zonular integrity is lost, resulting in iridodonesis. Lens subluxation, phacodonesis, and iridodonesis are relatively common findings in patients with MFS [21], WMS [54], and aniridia [55].

To better understand the relationship between the composition of the zonule and its biomechanics, the rheological properties of the zonule must be determined. Zonular fibers in human or bovine eyes are sufficiently large to manipulate directly (see Figure 1), and the forces generated when fibers are placed under tension can be measured using a probe coupled to a force transducer. Modeling the properties of the human zonule is crucial for studying MFS and the accommodative mechanism in general. However, to explore how zonule composition influences fiber rheology, a genetically tractable model like a mouse is required. Fortunately, numerous mouse models with targeted mutations in key microfibril components have been developed, and we have taken advantage of several such strains in our experiments (see below).

A significant challenge in using mice for biomechanical analysis is that the mouse zonule consists of approximately 30,000 delicate, translucent fibers, with widths ranging from 0.4 to 2.0 μm (see Figure 1D,G). While measuring the properties of individual fibers in the mouse model has not yet been possible, we have developed a technique called the “pull-up assay” to quantify the collective properties of zonular fibers. This technique has been described previously [56].

In brief, the pull-up assay begins by fixing an enucleated mouse eye with paraformaldehyde while maintaining a 15 mmHg positive internal pressure to prevent the collapse of the circumlental space and thus preserve zonular tension [35]. The eye is then glued, cornea down, to the base of a small buffer-filled chamber positioned on the weighing pan of a sensitive balance. The posterior part of the eye is removed to expose the back of the lens, to which a small probe is attached. Every 60 s, the probe is raised in 50 μm increments, and the weight of the chamber is recorded continuously. As the lens is lifted, the zonular fibers stretch, resulting in a decrease in the measured weight, which corresponds to the force applied to the fibers. This process continues until the zonular fibers break, allowing the chamber to return to its original position and the measured weight to return to the starting value.

**Figure 3:**
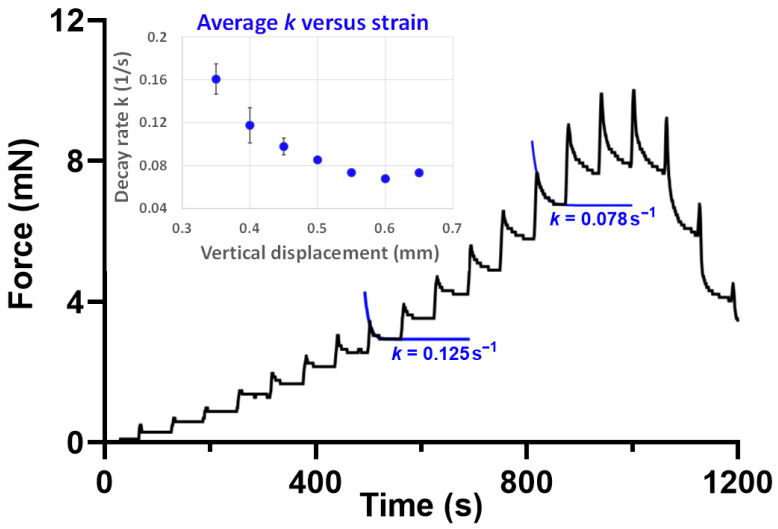
The viscoelastic behavior of the mouse zonule as measured using a pull-up assay (see [32,44,56] and text for methodological details). The lens is lifted from the eye in 50 μm steps (at a rate of 1 step per 60 s), causing a progressive increase in measured force as the zonular fibers stretch. In this example, fiber breakage begins when the lens is raised approximately 700 μm above its starting position. Each lens lift produces an instantaneous force increase, which then decays exponentially to a lower steady state with a time constant, *k*. This time-dependent decay is especially evident at higher displacement (strain) values. The inset shows the relationship between *k* and displacement distance.

This simple assay allows us to calculate several important properties, including the force required to break the fibers and the distance they stretch before breaking (displacement distance). At low strains, weight reduction occurs almost instantaneously when the lens is raised. However, at higher strains, the force response shows significant time-dependent behavior: an initial change in force that decays over more than 10 s to a new steady-state value (Figure 3). This strain- and time-dependent response is typical of viscoelastic materials and is characteristic of many biological molecules.

Additional information is needed to derive the elastic modulus of the fibers (i.e., Young’s modulus). Specifically, the force measurements must be normalized to the cross-sectional area of the fibers under test. While this measurement is relatively straightforward for large individual fibers (such as those of bovine or human eyes), it is challenging when working with populations of small caliber fibers, as in the mouse eyes used in the pull-up assays. Another difficulty is that tensile forces in the pull-up assay are not applied axially, causing the orientation of the fibers relative to the force vector to shift throughout the experiment. Additionally, as discussed, the zonular fibers behave not as simple springs but as viscoelastic structures with time-dependent properties.

To address these complexities, we measured the number and cross-sectional area of fibers in the mouse eye. We also developed a quasi-linear viscoelastic (QLV) model that accounts for both the viscoelastic behavior of the fibers and their fluctuating geometry [32]. The QLV model offers insights into the initial stiffness, final stiffness, decay time constant, and tensile strength (breaking force normalized to the cross-sectional area) of mouse eye zonular fibers.

### 5.1. The Wild-Type Zonule

**Figure 4:**
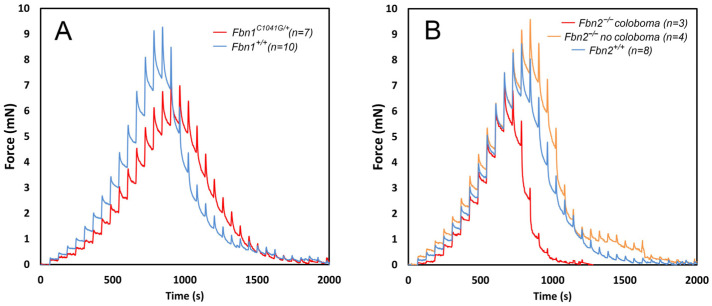
Biomechanical analysis of the ciliary zonule in wild-type and mutant mice measured using the pull-up assay (see [32,44,56] for methodological details). (**A**) The zonule is significantly weakened in mice heterozygous for the *FBN1 C1041G* mutation, and there is an accompanying increase in the displacement distance. (**B**) Compared to wild types, mice deficient in fibrillin-2 show reduced tensile strength and displacement distance in the presence of iris coloboma but not in its absence.

The mouse zonule emerges as a distinct structure toward the end of the first postnatal week, spanning the newly formed circumlental space. By one month of age, the zonule appears to be fully developed. Results from the pull-up assay show that zonular fibers at this stage typically rupture under a tensile force of 9.70 mN, with a maximum displacement of about 590 μm (Figure 4A). The initial stiffness, as calculated using the QLV model, is 0.235 MPa, which declines to a steady-state value of 0.097 MPa with a time constant of 16.05 s (Table 1). The tensile strength of wild-type zonular fibers is 0.952 MPa. These values closely align with those reported for human zonular fibers [57,58] and those of other species [59], likely reflecting the conservation of zonular composition and structural organization across species.

With age, the mouse zonule strengthens modestly. By 12 months, the breaking force rises to 11.65 mN, a 20% increase above the 1-month value (Table 1). There are corresponding age-dependent increases in stiffness and tensile strength.

### 5.2. Role of FBN1

Fibrillin-1 is the most abundant protein in zonular fibers [44]. Its contribution to zonular biomechanics can be explored by studying mice with mutant or null alleles of *Fbn1*. Germline knockout of *FBN1* causes perinatal lethality due to ruptured aortic aneurysms and impaired pulmonary function [60], precluding analysis of the zonule, which does not appear until the end of the first postnatal week. However, mice with a floxed *FBN1* allele [61] allow targeted deletion of *FBN1* in the NPCE [44], the layer that produces many structural components of the zonule, including fibrillin-1, fibrillin-2, and LTBP2 [32,43,44].

In *FBN1*-NPCE-conditional knockout mice, zonular fibers are still produced, likely due to compensatory upregulation of *FBN2* expression. However, the fibrillin-1-depleted zonule is significantly less robust than in wild-type mice, with a breaking force of 5.17 mN versus 9.68 mN [44]. This weakening reflects both a thinning of individual fibers and a reduction in their total number. Although the lens initially remains centered in the eye, segmental rupture of fibrillin-1-depleted zonular fibers begins at around 6 weeks of age, leading to subluxation. By three months, 100% of the fibrillin-1-deficient mice exhibit bilateral lens luxation [44].

One of the most extensively studied mouse models of MFS is the *FBN1^C1041G/+^* mouse, which models a human missense *FBN1* mutation and shows a relatively mild phenotype. These mice have normal lifespans but exhibit histological changes in the medial layer of the aorta and progressive skeletal abnormalities, such as kyphosis and rib overgrowth [62]. Unlike *FBN1*-conditional knockout mice, *FBN1^C1041G/+^* mice do not develop ectopia lentis. However, the ciliary zonule of *FBN1^C1041G/+^* mice is significantly weaker than in *FBN1^+/+^* controls (Figure 4A), with breaking forces of 7.13 ± 0.63 mN in mutants compared to 9.22 ± 1.64 mN in wild types (mean ± SD, *p* = 0.003) at 4 months of age (see also Table 1). Although the displacement distance was also greater in mutant mice, this difference did not reach statistical significance.

### 5.3. Role of FBN2

Fibrillin-2, the predominant fibrillin isoform in early development, remains a key component of the zonule in adult humans and cattle, ranking among the top ten most abundant glycoproteins in the zonular proteome of both species [17]. In human patients, mutations in *FBN2* lead to congenital contractural arachnodactyly (CCA), or Beals–Hecht syndrome, an autosomal dominant disorder with features that partially overlap with MFS. These include a marfanoid habitus, long fingers and toes, and kyphoscoliosis [63]. However, ectopia lentis is rarely reported in CCA patients.

We studied mice homozygous for an *FBN2* null allele [64]. Approximately half of the animals exhibited iridal phenotypes, such as coloboma, dyscoria, corectopia, and pseudopolycoria [65]. We assessed the mechanical properties of the zonule in fibrillin-2-deficient mice in the presence or absence of accompanying iridal abnormalities. In one-month-old animals with iris coloboma, the zonule was weaker than in *FBN2^+/+^* controls (Figure 4B), with a reduced breaking force of 7.09 ± 0.89 mN compared to 8.62 ± 2.76 mN in wild types. The decrease could be due to an observed reduction in the number of zonular fibers in the colobomatous region. *FBN2^−/−^* mice without coloboma had a breaking force of 9.57 ± 2.52 mN. The measured differences between groups did not reach statistical significance, probably because the sample sizes were relatively small (three–four eyes per group).

### 5.4. Role of MFAP2

MFAP2 is a small (≈20 kDa), cysteine-rich protein that associates with fibrillin-microfibrils [66] and is an abundant component of the ciliary zonule [40,41]. Although MFAP2’s function is not fully understood, MFAP2-deficient mice display a range of phenotypes, including increased body size, bleeding diathesis, bone abnormalities, and impaired injury responses [67]. MFAP2 binds directly to microfibrils, particularly in the bead region [68], and can interact with active TGFβ and BMPs [69]. Many traits in MFAP2 knockouts have been attributed to altered TGFβ signaling [70].

**Figure 5:**
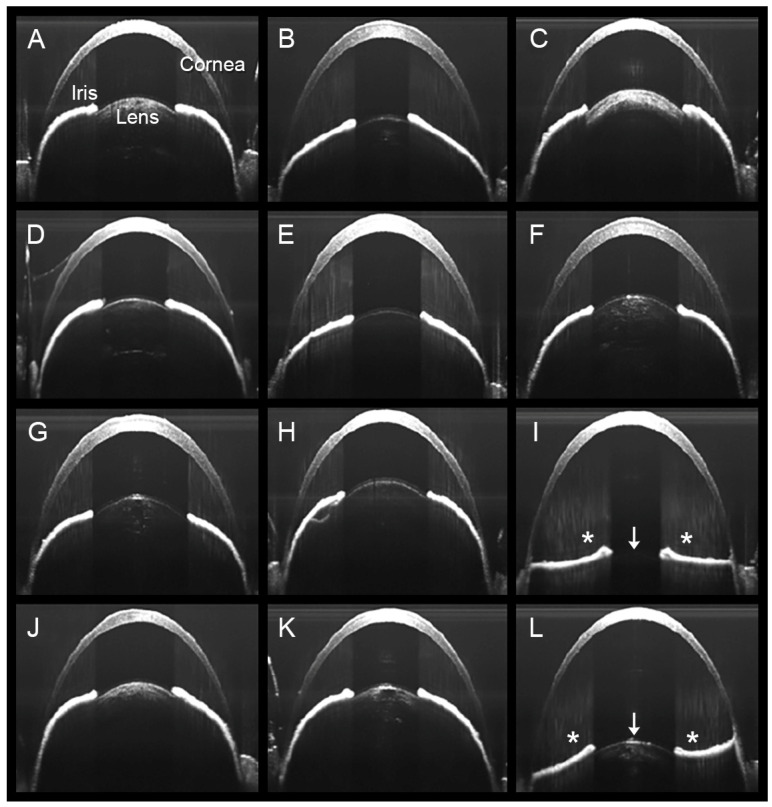
Lens luxation in *MFAP2^−/−^* mice. Panels (**A**–**L**) show the right eyes of twelve 18-month-old *MFAP2^−/−^* mice, imaged by optical coherence tomography (OCT) using methods described in [32,44]. The front surface of the lens (labeled in the first panel) is visible in the images. Note that in two of the twelve mice (shown in panels (**I**,**L**)), the lens is displaced backward in the eye (in the direction indicated by the arrows), resulting in flattening of the iris (*) and deepening of the anterior chamber (the space between the inner surface of the cornea and the front surface of the lens).

Though *MFAP2^−/−^* mice show no apparent ocular phenotype [65], we found their zonular mechanical properties to be significantly affected. At one month, the tensile strength of MFAP2-deficient fibers was reduced by approximately 50% compared to controls. This reduction persisted through at least 12 months, along with substantially decreased initial and final stiffness values (see Table 1). These findings indicate that, in the absence of MFAP2, zonular fibers become less stiff and more susceptible to breakage. Despite this persistent weakening, ectopia lentis was uncommon in *MFAP2^−/−^* mice (Figure 5). In the rare cases of lens dislocation, the effect was unilateral and did not occur until the animals were at least 18 months old. We never observed lens dislocation in age-matched heterozygous animals or their wild-type littermates.

### 5.5. Role of LTBP2

LTBP2 makes up approximately 10% of zonular mass in humans and cattle [40]. Super-resolution light microscopy and immuno-electron microscopy in mouse zonules reveal that LTBP2 is unevenly distributed along the zonular fibers [32]. In mice, *LTBP2* expression lags behind that of the fibrillins, beginning only at the end of the first postnatal week.

In humans, null mutations in *LTBP2* cause WMS3, a recessive syndrome characterized by short stature, brachydactyly, joint stiffness, and pulmonary and aortic stenosis [71]. Ocular symptoms include lens-induced myopia, microspherophakia, ectopia lentis, and glaucoma. Similarly, LTBP2-deficient mice develop ectopia lentis, typically by 4 months of age [32,72]. Unlike in human WMS3 patients, microspherophakia has not been reported in *LTBP2*-null mice.

Structural and mechanical assessments of *LTBP2^−/−^* mice show that at one month, their zonular fibers are indistinguishable from age-matched controls (Table 1). However, tensile strength declines subsequently, with fibers showing overt rupture by two months, subluxation by four months, and complete lens luxation by six months [32].

### 5.6. Role of LOXL1

LOXL1 is an enzyme that catalyzes the cross-linking of tropoelastin monomers during elastogenesis [46]. It is expressed strongly in mechanically stressed tissues like the lungs and aorta [73] and interacts closely with elastic fibers [74]. Variants of *LOXL1* are associated with exfoliation syndrome, a condition characterized by the accumulation of insoluble fibrils in the eye and other tissues [75]. Notably, exfoliation syndrome increases the risk of lens dislocation due to compromised zonular fibers and exfoliation glaucoma, an aggressive type of glaucoma.

*LOXL1*-null mice exhibit skin laxity, enlarged alveoli, vascular abnormalities, and pelvic organ prolapse; these effects vary somewhat depending on genetic background [76]. The ocular manifestations include lens abnormalities, disruption of the blood–aqueous barrier [77], and mildly elevated intraocular pressure in certain strains [78]. Additionally, these mice have increased anterior chamber depth and variable changes in the dimensions of Schlemm’s canal, along with enlarged optic nerves and a stiffening of the peripapillary sclera [79].

**Figure 6:**
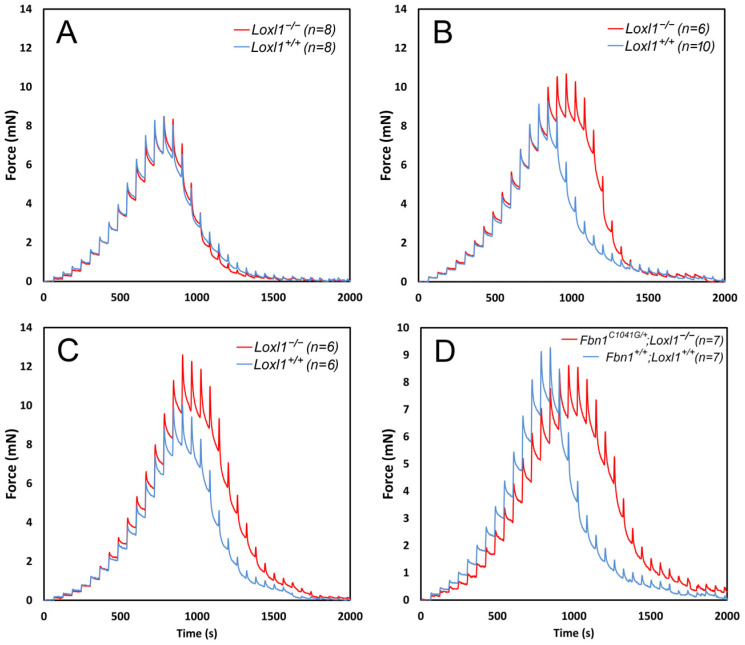
The absence of LOXL1 leads to age-dependent strengthening of the ciliary zonule (see [32,44,56] for methodological details). (**A**) At 1 month of age, the biomechanical properties of the zonule in *LOXL1^+/+^* and *LOXL1^−/−^* mice are indistinguishable. (**B**) By four months of age, the displacement distance and the breaking force increase in *LOXL1*-deficient mice compared to the wild type. (**C**) By 6 months, differences in breaking force and displacement distance between the genotypes have become statistically significant. (**D**) One-month-old mice heterozygous for the *C1041G FBN1* mutation usually exhibit decreased breaking force and increased displacement distance (see Figure 4A). Zonular breaking force (but not displacement distance) is restored in compound *FBN1^C1041G/+^*; *LOXL1^−/−^* mutants.

LOXL1 has been localized to the zonule via mass spectrometry [40] and immunocytochemistry [45], which is intriguing given the nominal absence of elastin from the zonule. Although elastin is the primary known substrate for LOXL1, other proteins, such as fibrillin-1, could theoretically serve as substrates. To explore this, we examined the mechanical properties of the zonule in *LOXL1^−/−^* mice (Figure 6).

At one month of age, the zonules of *LOXL1*-null mice were indistinguishable from those of wild-type mice (Figure 6A). By 4 months (Figure 6B), however, the zonules of the mutant mice had strengthened relative to wild types, with the difference reaching statistical significance by six months (Figure 6C). At six months, the zonular breaking force in *LOXL1^−/−^* mice was 12.60 ± 2.35 mN compared to 10.02 ± 0.81 mN in wild types. As noted earlier, mice heterozygous for the *FBN1 C1041G* mutation have weakened zonules (see Figure 4A). This phenotype was rescued in *FBN1/LOXL1* compound mutants (Figure 6D). In animals homozygous for a *Loxl1*-null mutation and heterozygous for the *FBN1 C1041G* mutation, the zonular breaking force was statistically indistinguishable from wild-type controls (9.22 ± 1.64 mN (wild type) versus 8.65 ± 0.89 mN (compound mutant)).

The unexpected age-dependent *increase* in breaking force in *Loxl1*-null mice implies that LOXL1 may not serve to cross-link and strengthen zonular components as previously thought. Preliminary data indicate that *Fbn1* expression is elevated in the eyes of *LOXL1^−/−^* mice, suggesting a possible gene regulatory function for LOXL1 if these findings are confirmed. We note that others have reported broad transcriptional changes in several organs of *LOXL1*-deficient mice, with significant effects on the expression of cell cycle and immune response genes [80].

## 6. Conclusions

**Figure 7:**
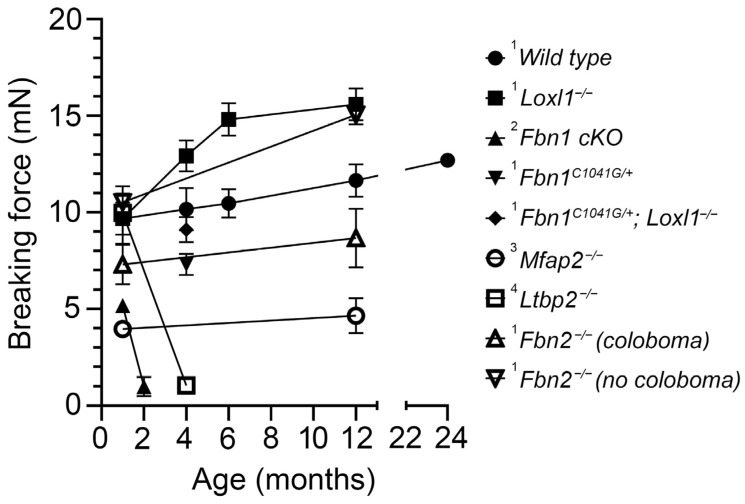
The effect of targeted manipulation of zonular components on the breaking force of the mouse zonule (see text for details). The strength of the wild-type zonule increases modestly with time. Targeted deletion of *FBN1* or *LTBP2* results in catastrophic failure of the zonule by 2 and 4 months, respectively. Colobomatous *FBN2*-null mice, *MFAP2*-null mice, and *FBN1^C1041G/+^* mice have weakened zonules, but their lenses do not dislocate. Mice deficient in *LOXL1* or non-colobomatous *FBN2*-null mice have stronger zonules than wild types. The phenotype of mice harboring compound *FBN1^C1041G^*; *LOXL1^−/−^* mutations is intermediate between that of mice carrying the individual mutations. Data are from ^1^ this paper; ^2^ [44]; ^3^ [56]; ^4^ [32].

The eye is notably affected in MFS and WMS, two conditions linked to microfibril dysfunction. The zonule offers a unique model for studying how disease-causing mutations impact microfibril composition, structure, and especially rheological properties. We have developed a pull-up assay technique to quantify the collective mechanical properties of the thousands of fibers that make up the mouse zonule. Using the mouse model, we examined the biomechanical effects of targeting key structural or enzymatic components (summarized in Figure 7 and Table 1).

Several caveats apply when interpreting these data. One assumption is that mechanical changes in zonular properties stem from the absence of the targeted component, presupposing no compensatory changes in the zonule, which is unlikely. For instance, we have shown previously that the lack of fibrillin-1 leads to overexpression of fibrillin-2 in the zonule and that the converse also occurs [44]. Therefore, caution is required when attributing observed changes in zonular properties solely to the loss of a single targeted gene.

We also assume that the overall composition of the mouse zonule is similar to that of humans and cattle. However, while the human and bovine proteomes have been reported [39,40,41], similar studies are lacking for the mice zonule, primarily because of its comparatively smaller size.

Another limitation is our lack of a precise structural model of the zonule, particularly regarding the length of individual microfibrils. Whether single microfibrils extend the entire length of a zonular fiber (more than a millimeter in some species) or if a fiber comprises a series of shorter, overlapping microfibrils affects our understanding of how fibers stretch and how newly synthesized components are integrated.

An additional challenge has been accounting for the elasticity of the zonule in light of the properties of its constituent microfibrils. The stiffness of individual microfibrils, approximately 100 MPa [9], is significantly higher than that of the zonule overall—a curious phenomenon. An apt analogy would be gathering a handful of fishing lines and finding that the resulting bundle of stiff fibers behaves like a rubber band. While it is conceivable that a fiber composed of stiff microfibrils could stretch through shear interactions, this behavior has proven difficult to demonstrate experimentally.

Megill et al. evaluated several structural models for microfibril-based fibers in the hydromedusa *Polyorchis penicillatus* [12]. They ultimately opted for a model that featured parallel bundles of continuous, relatively elastic microfibrils. The investigators reasoned that previous measurements of individual microfibrils might have been influenced by non-linear regions of the stress–strain curve, leading to anomalously high stiffness values.

Regarding the MFS phenotype, we previously demonstrated that conditional deletion of *FBN1* in mice leads to ectopia lentis and other MFS-relevant ocular phenotypes, such as cataracts and axial elongation [44]. In the current study, we found that mice heterozygous for the MFS *C1041G* mutation do not exhibit lens luxation (at least not by 4 months of age) despite a marked reduction in breaking force and an increase in displacement distance. Consequently, the zonular fibers become hyperextendable, mimicking the fibers in MFS patients, which tend to hyperextend rather than rupture, leading to progressive lens decentration [21].

Interestingly, zonular integrity is largely restored in compound mutants (*FBN1^C1041G/+^; LOXL1^−/−^*). Similarly, the ectopia lentis phenotype in *LTBP2*-null mice [32,72] can be rescued by overexpressing the structurally related protein LTBP4 [81]. Studies of this sort are encouraging because they suggest potential strategies for alleviating some of the ocular symptoms of MFS and related diseases. Whether a gene therapy approach is viable in adult MFS patients may depend on the extent of ongoing microfibril synthesis. The observation that the zonule of wild-type mice strengthens with time supports the idea of continuous, though possibly slow, microfibril production. This finding is also consistent with reports that mRNA transcripts encoding fibrillin-1 and other zonule proteome components are detectable in the NPCE cells of mice until at least one year of age and likely older.

The strength of fibers at one month of age is not entirely predictive of subsequent lens dislocation risk (see Figure 7). For instance, MFAP2-deficient mice exhibit a 50% reduction in breaking force at one month but seldom show lens dislocation (and only then in aged animals). In contrast, the zonules of LTBP2-deficient mice are initially indistinguishable from wild types, but thereafter weaken, leading to lens dislocation by four months. Thus, lens dislocation is not an inevitable result of decreased tensile strength. We hypothesize that the absence of proteins such as LTBP2 may make the fibers more prone to proteolysis, but more experiments will be needed to test this notion directly.

Finally, the zonular fibers demonstrate clear strain-dependent viscoelasticity. It is worth considering whether the viscoelastic properties are tuned to support the accommodative mechanism in humans. During near focus, the ciliary muscle contracts, drawing the ciliary processes inward and forward, which stretches the zonular fibers in the pars plana region of the eye. Given their stiffness, the initial force required to extend the pars plana zonular fibers would be high. However, this decreases over a few seconds, reducing the effort required for the ciliary muscle to sustain near focus. Moreover, given that smooth muscles also undergo stress relaxation upon contraction, this raises the intriguing possibility that the two relaxation mechanisms could be synchronized. As we gain further insights into zonular properties, such as resting strain and detailed internal anatomy, we can construct more accurate models of the human accommodative mechanism.

## Figures and Tables

**Table 1:** Summary of the rheological properties of zonular fibers in wild-type and mutant mice. EL—ectopia lentis. Data from ^1^ this paper; ^2^ [56]; ^3^ [32]; ^4^ [44].

Genotype (Age)	Breaking Force (mN)	Final Displacement (mm)	Zonular Appearance	EL	Initial Stiffness G_0_ (MPa)	Final Stiffness G_∞_ (MPa)	Time Constant (s)	Tensile Strength σ *_ f _* (MPa)	*n*
^1^ *LOXL1^+/+^* (1 M)	9.67 ± 1.30	0.64 ± 0.05	Normal	No					8
^2^ *MFAP2^+/+^* (1 M)	8.57 ± 0.35	0.63 ± 0.04	Normal	No	0.234	0.093	16.3	0.961	6
^3^ *LTBP2^+/+^* (1 M)	9.21 ± 0.43	0.66 ± 0.05	Normal	No	0.237	0.110	15.8	0.943	4
^1^ *LTBP2^+/+^* (4 M)	10.51 ± 0.87	0.89 ± 0.06	Normal	No					4
^4^ C57BL/6J (1 M)	9.68 ± 0.3	0.54 ± 0.015	Normal	No					3
^1^ *LOXL1*^+/+^ (4 M)	10.16 ± 1.09	0.70 ± 0.05	Normal	No					10
^1^ *LOXL1^+/+^* (6 M)	10.46 ± 0.97	0.74 ± 0.03	Normal	No					6
^4^ C57BL/6J (12 M)	11.65 ± 0.94	0.75 ± 0.06	Normal	No					3
^2^ *MFAP2^+/+^* (12 M)	11.85 ± 1.31	0.80 ± 0.04	Normal	No	0.198	0.074	17	1.41	6
^1^ *LOXL1^−/−^* (1 M)	9.67 ± 0.83	0.68 ± 0.08	Normal	No					8
^1^ *LOXL1^−/−^* (4 M)	12.92 ± 0.96	0.80 ± 0.10	Normal	No					6
^1^ *LOXL1^−/−^* (6 M)	14.81 ± 1.63	0.84 ± 0.08	Normal	No					6
^1^ *LOXL1^−/−^* (12 M)	15.59 ± 0.09	0.82 ± 0.02	Normal	No					2
^4^ *FBN1* cKO (1 M)	5.17 ± 0.20	0.525 ± 0.025	Depleted fibers	No					3
^4^ *FBN1* cKO (2 M)	0.98 ± 0.49	0.72 ± 0.18	Zonular dehiscence	Yes					3
^1^ *FBN1^C1041G/+^* (4 M)	7.30 ± 0.55	0.77 ± 0.04	Normal	No					7
^1^ *FBN1^C1041G/+^*; *Loxl1^−/−^* (4 M)	9.10 ± 0.65	0.83 ± 0.05	Normal	No					5
^2^ *MFAP2^−/−^* (1 M)	3.96 ± 0.38	0.80 ± 0.04	Normal	No	0.063	0.021	3.8	0.078	5
^2^ *MFAP2^−/−^* (12 M)	4.65 ± 0.89	0.92 ± 0.07	Normal	Rare	0.017	0.025	12.9	0.505	5
^3^ *LTBP2^−/−^* (1 M)	3.97 ± 0.30	0.51 ± 0.02	Normal	No	0.235	0.066	18.0	0.405	3, 4
^1^ *LTBP2^−/−^* (4 M)	1.03 ± 0.19	0.83 ± 0.11	Zonular dehiscence	Yes					3
^1^ *FBN2^−/−^* (1 M) coloboma	7.30 ± 1.02	0.58 ± 0.04	Keyhole iris	No					3
^1^ *FBN2^−/−^* (1 M) *no coloboma*	10.52 ± 0.83	0.67 ± 0.07	Normal	No					4
^1^ *FBN2^−/−^* (12 M) *coloboma*	8.67 ± 1.52	0.66 ± 0.07	Keyhole iris	No					4

## Data Availability

The raw data supporting the conclusions of this article will be made available by the authors on request.
